# A Forensic Medical Evaluation of Cases Developing Permanent Sequelae Following Traffic Accident-Related Ocular Injuries

**DOI:** 10.3390/diagnostics16182911

**Published:** 2026-09-09

**Authors:** Ömer Turan, İsmail Altin, Fehim Esen

**Affiliations:** 1 Department of Forensic Medicine, School of Medicine, Istanbul Medeniyet University, Istanbul 34722, Türkiye; dromerturan@gmail.com; 2 Department of Ophthalmology, School of Medicine, Istanbul Medeniyet University, Istanbul 34722, Türkiye

**Keywords:** eye injuries, traffic accidents, optic atrophy, intracranial hemorrhages, visual acuity, disability evaluation

## Abstract

**Background/Objectives**: To identify factors associated with post-healing visual impairment and describe clinical characteristics in patients with traffic-related ocular trauma undergoing forensic evaluation. **Methods**: We retrospectively reviewed 75 eligible cases with traffic accident-related ocular injury assessed at a university hospital Forensic Medicine Department between 2019 and 2024, after completion of treatment. Collected variables included demographics, accident and trauma type, ophthalmic and neurologic findings, best-corrected visual acuity (converted to LogMAR), and disability ratings. **Results**: Of 75 cases, 58.7% were male; in-vehicle accidents were most frequent (45.3%). Visual status at evaluation was: no impairment 50.7%, mild 13.3%, moderate 9.3%, and severe or total loss 26.7%. Exploratory omnibus analyses showed associations of visual-impairment severity with optic atrophy (*p* < 0.001) and intracranial bleeding (*p* = 0.007). Disability percentages increased stepwise with worsening visual impairment. **Conclusions**: In this post-healing forensic cohort, severe visual impairment was associated with optic atrophy and intracranial bleeding, and disability percentages rose with impairment severity. Standardized visual metrics and targeted neurologic assessment may be useful components of post-healing disability evaluation; however, their effects on the consistency and reliability of disability determinations were not directly assessed in this study.

## 1. Introduction

Traumatic eye injuries are one of the leading causes of sight loss throughout the world in general, and can lead to serious and permanent outcomes especially in young and reproductive age individuals. As a leading cause of sight loss in the young and active population, traumatic eye injuries constitute a significant public health problem on a global scale [1]. Global Burden of Disease Study 2019 estimates indicate that approximately 59.9 million incident cases of eye injury occurred worldwide in 2019 [1]. Traffic accidents constitute a significant mechanism for ocular trauma [2]. Traumas in the eye, head, and face region present with various clinical conditions such as corneal abrasion, hyphema, lens dislocation, retinal detachment, and globe rupture, and in some cases can progress as far as permanent sight loss [2,3].

Traffic accidents are among the primary causes of traumatic eye injuries and in developing countries, this rate is seen to be even higher. Among vehicle occupants, ocular injury may result from airbag deployment or direct contact with vehicle structures, while seatbelt use has been associated with a lower risk of ocular injury [4]. Motorcycle accidents can result in more serious eye lesions such as open globe injuries and orbital fractures due to riders not wearing a helmet [5]. Traffic-related ocular injuries can result in persistent visual impairment, particularly in patients with severe posterior-segment involvement. Seatbelt use has been associated with less severe ocular injuries and better visual outcomes following motor vehicle accidents with airbag deployment [6,7].

Although studies specifically comparing ocular injuries by traffic-accident type remain limited, recent prospective data show that road traffic accidents account for a substantial proportion of ocular trauma presentations and that these injuries encompass varied patterns of tissue involvement, management requirements, and visual outcomes [8]. In motorcycle crashes, helmet use and helmet type may also influence the associated facial and zygomatic–orbital fracture pattern [5].

The most common injuries in within-vehicle accidents include corneal abrasion, hyphema, and vitreous hemorrhage, while orbital fracture and open globe injuries are seen at a higher rate in outside-the-vehicle accidents [9,10]. In motorcycle accident ocular injuries, not using protective equipment is predominant as a significant risk factor increasing the severity of the injury [11]. It has been shown in some studies that the incidence of ocular trauma is significantly higher in males in motorcycle accidents, and this could be associated with both the driver profile and behavioral risk factors [12]. Statistically significant correlations between accident types and trauma localization, severity, and accompanying head trauma have been shown in various retrospective series [13,14].

The aim of this retrospective study was to characterize traffic-related ocular trauma among patients who, after completion of treatment, presented to the Forensic Medicine Department for evaluation of permanent damage. We recorded the distribution of permanent sequelae affecting visual function and the corresponding disability ratings. Demographic characteristics, accident type (in-vehicle, out-of-vehicle, motorcycle), ophthalmic findings, and relevant neurologic/radiologic findings were analyzed to identify associated factors for visual sequelae. To our knowledge, this is among the few studies from Türkiye to examine permanent sequelae after traffic-related ocular trauma from a forensic medical perspective, and one of the first to focus specifically on the relationship between visual loss and disability ratings. Clarifying which injury patterns are linked to specific sequelae by accident type may inform prevention strategies and facilitate earlier recognition and documentation of permanent damage.

## 2. Materials and Methods

**Figure 1:**
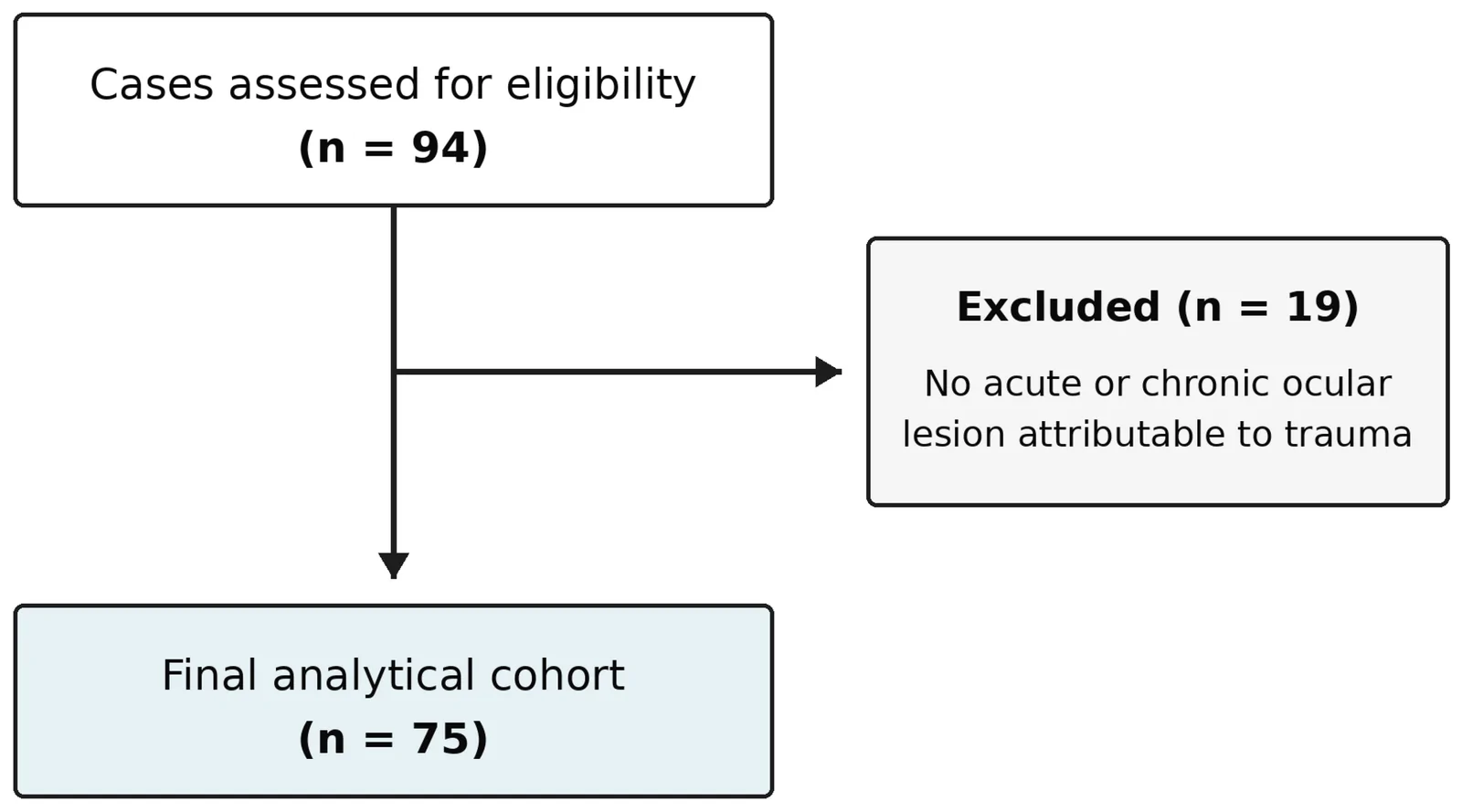
Patient selection flow diagram.

In this retrospective descriptive study, an examination was made of the forensic records of individuals who presented at the Forensic Medicine Department of a university hospital between 2019 and 2024 because of traffic accident-related ocular injury. During the study period, 94 cases referred for forensic evaluation following traffic accident-related ocular trauma were assessed for eligibility. Nineteen cases were excluded because no acute or chronic ocular lesion attributable to trauma was identified. The final analytical population therefore comprised 75 cases (Figure 1). The individuals included in the study were those who had sustained eye trauma and for whom a forensic medical report had been produced for evaluation of permanent damage after completion of all the medical treatment processes. Completion of treatment and stabilization of the ocular condition were determined by reviewing the previous medical records together with the findings of the most recent ophthalmological examination. Persistent findings documented at this final post-treatment examination were used for the forensic disability assessment. Cases were excluded from the study if they did not have any ocular trauma, had incomplete data in the records, or had developed ocular damage for reasons other than trauma.

Case information was abstracted from forensic medical examination reports, hospital records, ophthalmology consultation notes, imaging studies, and, when necessary, local forensic unit reports. For each case, we recorded age, sex, accident type (in-vehicle, out-of-vehicle, motorcycle), type of ocular trauma, permanent visual impairment attributable to the trauma, concomitant orbital fracture, intracranial hemorrhage, and neurologic involvement. The study included individuals with ocular trauma who underwent final forensic evaluation for permanent sequelae at least six months after the traffic accident, following completion of treatment and clinical stabilization of the ocular condition. In patients with unilateral ocular injury, trauma-related visual impairment was classified according to the best-corrected visual acuity of the injured eye. In patients with bilateral ocular injury, the best-corrected visual acuity of the more severely affected eye was used for classification. Trauma-related visual impairment was categorized using prespecified study-specific best-corrected visual acuity ranges as normal (0.8–1.0), mild (0.4–0.7), moderate (0.1–0.3), or severe/total (<0.1, including counting fingers, hand motion, light-perception, and no-light-perception levels). These categories were used to describe visual outcomes in the present forensic cohort and should not be interpreted as the standard WHO visual-impairment categories. Visual acuity values for the right and left eyes were converted separately to LogMAR. For the continuous patient-level LogMAR variable, the injured-eye value was used in unilateral cases, whereas the value of the more severely affected eye was used in bilateral cases. The arithmetic mean of the two monocular LogMAR values was not used. Pre-existing visual impairment was assessed by reviewing pre-accident ophthalmologic and other available medical records. When pre-existing impairment was documented, the pre-injury visual status was used as the baseline, and only the additional visual impairment attributable to the traffic accident was included in the trauma-related outcome classification. Cases in which ocular impairment was attributable to a non-traumatic cause were excluded.

Decimal visual acuity values were converted to LogMAR using the formula −log10 (decimal visual acuity). For visual acuity levels that could not be expressed using Snellen notation, the following numerical assignments were used: counting fingers, 1.9 LogMAR; hand motion, 2.3 LogMAR; light-perception, 2.7 LogMAR; and no-light-perception, 3.0 LogMAR. These assignments were based on previously published low-vision conversion conventions [15].

Total-body disability scores attributable to ocular trauma were calculated in accordance with Türkiye’s Regulations on Disability Assessment of Adults (Erişkinler İçin Engellilik Değerlendirmesi Hakkında Yönetmelik), published in the Official Gazette (No. 30692; 20 February 2019). Disability ratings were derived from the visual-system criteria specified by this national framework, which formed the legal basis for forensic reporting [16]. Disability percentages attributable to ocular trauma were calculated according to the visual-system criteria of Türkiye’s Regulations on Disability Assessment of Adults [16]. Best-corrected visual acuity values for the right eye, left eye, and binocular vision were converted to visual acuity scores using the regulatory conversion table. The functional visual acuity score was calculated as [(3 × binocular score) + right-eye score + left-eye score]/5, corresponding to weights of 60%, 20%, and 20%, respectively. When a visual-field defect was present, the functional visual-field score was calculated using the same weighting; otherwise, the visual-field score was accepted as 100. The functional vision score was calculated by multiplying the functional visual acuity and visual-field scores and dividing the result by 100. Additional permanent ocular sequelae, such as diplopia, qualifying eyelid or lacrimal dysfunction, nystagmus with compensatory head posture, and manifest strabismus, were incorporated using the adjustment values specified in the regulation. The visual-system impairment rate was calculated as 100 minus the functional vision score. When this rate was ≤50, it was accepted directly as the disability percentage; values > 50 were converted using the formula: disability percentage = [(visual-system impairment rate − 50) × 0.8] + 50. Disability percentages were jointly determined by forensic medicine and ophthalmology specialists during preparation of the final medico-legal report, based on the ophthalmological examination and consultation findings. Trauma-related causation was established by reviewing the pre-accident and post-accident medical records together with the final ophthalmological examination; pre-existing or non-traumatic impairment was excluded, and only additional permanent impairment attributable to the traffic accident was incorporated into the disability assessment.

### Statistical Analysis

Data were analyzed using IBM SPSS Statistics for Windows, version 19.0 (IBM Corp., Armonk, NY, USA). Normality was assessed with the Shapiro–Wilk test. Descriptive statistics are reported as mean ± standard deviation for continuous variables and number (*n*) and percentage (%) for categorical variables. The primary analysis was descriptive. All comparisons according to accident type and visual-impairment category were secondary, univariable, and exploratory; no formal hierarchy of prespecified confirmatory hypotheses was defined, and no multivariable model was performed. Bonferroni adjustment was applied only to post hoc pairwise comparisons following an omnibus test. No global multiplicity adjustment was applied across the separate exploratory comparisons; therefore, the reported *p* values are nominal and should be interpreted cautiously, particularly when close to 0.05. Associations between categorical variables were evaluated using Pearson’s chi-square test when the expected-frequency assumptions were satisfied. When these assumptions were not met, Fisher’s exact test was used for 2 × 2 tables and the Fisher–Freeman–Halton exact test with Monte Carlo estimation was used for larger contingency tables. For comparisons of measured data across more than two independent groups, one-way ANOVA was used for normally distributed variables and the Kruskal–Wallis test for non-normal variables. Type 1 error level was accepted as α = 0.05. For the principal clinically relevant associations, effect sizes were expressed as unadjusted odds ratios (ORs) with 95% confidence intervals (CIs) after dichotomizing the visual outcome as severe/total impairment versus all other visual-impairment categories.

## 3. Results

Seventy-five cases were evaluated (58.7% male, 41.3% female; mean age 32.7 ± 15.8 years). Accident types were in-vehicle (45.3%), out-of-vehicle (24.0%), and motorcycle (30.7%). Demographic, accident-related, and medico-legal characteristics are presented in Table 1. The mean patient-level LogMAR visual acuity was 0.72 ± 1.03 among the 74 patients with available visual-acuity data. Post-healing ocular and associated clinical findings are summarized in Table 2. Eyelid/periorbital injuries (41.3%) and ocular motility restriction (32.0%) were the most frequent findings after the accident. Optic atrophy was present in 26.7% of patients and neuro-ophthalmic involvement in 9.3%.

Associations of accident type with demographic and general clinical characteristics and with detailed ocular examination findings are presented in Table 3 and Table 4, respectively. Motorcycle accidents involved predominantly males (91.3% vs. 8.7% females; *p* < 0.001). No significant association was observed between accident type and visual acuity, neuro-ophthalmic involvement, orbital fracture, or the major ocular and periocular finding groups (all *p* > 0.05).

Visual status at evaluation was: no impairment 50.7%, mild 13.3%, moderate 9.3%, and severe/total 26.7%. Disability scores differed significantly across the visual-impairment categories (omnibus *p* < 0.001). Bonferroni-adjusted post hoc comparisons indicated that disability scores were higher in the severe/total group than in the no-impairment and mild-impairment groups, whereas the moderate group did not differ significantly from the other groups. No significant associations were observed between demographic variables and visual-impairment severity (all *p* > 0.05). Exploratory omnibus analyses showed overall associations of visual-impairment severity with optic atrophy (*p* < 0.001) and intracranial bleeding (*p* = 0.007). Nominal associations were also observed for eyelid/periorbital lesions (*p* = 0.024) and eye movement findings (*p* = 0.045). Given the number of comparisons and the absence of a global multiplicity adjustment, these latter findings should be interpreted cautiously. No other ocular findings showed significant associations with visual-impairment severity (*p* > 0.05; Table 5). To quantify the magnitude of the principal associations, severe/total visual impairment was compared with all other visual-impairment categories. The odds of severe/total visual impairment were higher among patients with optic atrophy (11/20 vs. 9/55; OR = 6.25, 95% CI: 2.01–19.42) and among those with intracranial bleeding (8/12 vs. 12/63; OR = 8.50, 95% CI: 2.19–32.95). Of the 75 patients, 65 had unilateral ocular injuries and 10 had bilateral ocular injuries.

## 4. Discussion

In this medico-legal cohort of traffic accident-related ocular injuries, approximately half of the patients had no visual impairment, whereas more than one in four presented with severe/total impairment at post-healing evaluation. The two findings associated with advanced impairment were optic atrophy and intracranial bleeding, underscoring that both optic-nerve damage and concomitant head injury were associated with worse post-healing visual status relevant to disability determinations. Clearly defined visual-acuity categories and LogMAR values may provide a standardized framework for forensic reporting; however, their effects on objectivity and reproducibility were not directly assessed in this study. To our knowledge, few studies have reported post-healing medico-legal assessments integrating visual-acuity outcomes with disability ratings after traffic-related ocular trauma; within Türkiye, such delayed, forensic-oriented evaluations remain uncommon [17].

Prior work has reported that roughly 25% of ocular traumas result in permanent visual loss [3], and contemporary clinical evidence continues to identify ocular trauma as an important cause of permanent vision loss [18]. Road traffic accidents have also remained a leading mechanism of ocular and oculofacial trauma in recent clinical and meta-analytic studies [19,20]. Our findings are consistent with this burden and with the literature noting that nearly one third of individuals with ocular trauma develop permanent anatomic damage, typically involving the anterior or posterior segment [3]. In the present cohort, anterior-segment involvement was observed in 20% and posterior-segment involvement in 13.3%. Anatomic profile matters: retinal or choroidal detachment and traumatic optic neuropathy have been associated with severe visual impairment or worse visual outcomes, while optic nerve involvement has been associated with adverse visual outcomes [18,19,20]. This pattern—also reflected in our data—helps distinguish injuries that primarily impair comfort or motility from those that directly depress visual acuity, the latter being decisive for medico-legal disability.

When clinical characteristics were examined by accident type, males were disproportionately represented among motorcycle accidents. Similar male predominance and potentially serious ocular outcomes have been reported in recent motorcycle- and road traffic-related ocular-trauma studies [21,22,23]. Nevertheless, no clear association was observed between accident type and visual-impairment severity in our cohort. Although the motorcycle subgroup had numerically better mean visual acuity, this finding should be interpreted cautiously and regarded as hypothesis-generating. Previous studies indicate that helmet use, particularly in combination with a visor, may reduce the severity of ocular and periocular injuries [22,24]. Because protective-equipment use was not recorded in the present study, its potential effect could not be evaluated. Future studies should prospectively document helmet, visor or goggle, and seatbelt use to clarify mechanism–outcome relationships [22,23,24].

Although the differences were not statistically significant, eyelid and periorbital lesions were proportionally most frequent in motorcycle accidents, whereas eye movement problems, including diplopia, were proportionally most frequent in out-of-vehicle accidents. The predominance of ophthalmoplegic findings in out-of-vehicle accidents is anatomically plausible because orbital fractures may be accompanied by diplopia and restricted extraocular movements [4,13,25,26]. Recent road-traffic and orbital-trauma series have likewise identified eyelid laceration, periorbital swelling or ecchymosis, and orbital wall fractures as frequent components of the injury spectrum [27,28]. Nevertheless, no clear association was observed in our cohort between accident type and orbital fracture, neurologic involvement, or posterior-segment findings. This may reflect the small subgroup sizes and unmeasured heterogeneity in protective-equipment use.

Visual impairment in traffic-related ocular trauma was associated not only with direct ocular damage but also with neuro-ophthalmic and central nervous system involvement [29,30,31,32]. Severe or total visual impairment was associated with optic atrophy in the separate dichotomized analysis, consistent with evidence that traumatic optic nerve injury and avulsion commonly produce poor or permanent visual outcomes [17,29,30]. The observed association between severe impairment and intracranial bleeding may indicate concomitant injury to the central visual pathways or cortical visual-processing regions; however, the present data cannot establish the precise anatomical mechanism [31,32]. From a clinical perspective, persistent visual loss after traffic trauma warrants neurologic and neuro-ophthalmologic assessment together with appropriate cranial and orbital imaging, even when external ocular findings appear superficial [29,30,31,32]. The nominal association between eye movement findings and visual-impairment severity reflected lower frequencies of severe acuity loss among patients with motility abnormalities. Nevertheless, post-traumatic oculomotor abnormalities may cause diplopia and substantial functional symptoms without necessarily producing a comparable loss of visual acuity [32,33].

The male sex was overrepresented among motorcycle-related eye injuries in our cohort. This finding is consistent with the marked male predominance reported in previous studies of motorcycle-associated ocular trauma [21,34]. Broader motor-vehicle collision research has likewise demonstrated a substantially greater male predominance among motorcyclists than among other road-user groups, supporting differences in exposure according to road-user type [35]. Female patients constituted similar proportions of the in-vehicle and out-of-vehicle groups and were markedly underrepresented in the motorcycle group. However, because seat position, seatbelt use, helmet use, and other protective factors were not recorded, these observations should not be attributed to these factors. No direct association between sex and impairment severity was detected. This is compatible with broader trauma data showing sex-related differences in injury patterns but no difference in adjusted outcomes, although these findings cannot be directly extrapolated to visual outcomes [36]. Larger studies with prospective recording of exposure and protective-equipment use are needed to clarify whether sex-specific differences exist.

Objective disability measurement is critical in compensation determinations. In our cohort, disability ratings increased stepwise with impairment severity. However, because visual acuity is a direct component of the regulatory disability calculation, this relationship is partly inherent to the scoring system and should not be interpreted as an independent clinical finding. Previous studies indicate that visual impairment may result in substantial disability, particularly when the optic nerve, ocular motor system, or central visual pathways are involved [30,31,32,33,37]. Consistent with this, cases with optic atrophy or intracranial bleeding had markedly higher disability ratings. These findings support a comprehensive assessment after completion of treatment and clinical stabilization, incorporating ophthalmologic and neurologic examinations together with appropriate radiologic evaluation rather than an eye-only approach [31,32]. Standardized assessment of visual acuity and ocular motility may facilitate a structured forensic evaluation [31,32,33]. Recommendations regarding standardized visual assessment, neurologic evaluation, and imaging represent clinical implications derived from the observed associations and existing literature; their effects on diagnostic yield, inter-report consistency, and medico-legal outcomes were not directly evaluated in this study. Because imaging modalities and their diagnostic yield were not compared, the present study cannot support routine brain and orbital MRI for all patients or define a universal imaging protocol; imaging decisions should instead be guided by the clinical and neuro-ophthalmologic findings. Within Türkiye, disability determinations should be based on the Regulation on Disability Assessment for Adults and its visual-system criteria, where applicable [16]. Our series adds to the limited Türkiye-based forensic evidence and highlights the need for multicenter datasets capturing protective-equipment use and time-to-assessment to refine risk estimates and strengthen causal inference. Although the regulatory calculation incorporates certain non-acuity sequelae—including diplopia, manifest strabismus, nystagmus with compensatory head posture, and qualifying eyelid or lacrimal dysfunction—visual-acuity categories alone do not capture the full functional and cosmetic impact of ocular motility and periocular disorders. Consequently, some patient-reported limitations may not be fully reflected in the disability percentage. Because this disability-rating system is specific to Türkiye’s legal framework, the resulting disability percentages may not be directly applicable in countries that use different legal or regulatory criteria.

### Limitations

This retrospective study relied on chart abstractions and forensic documents; some subgroups (e.g., posterior-segment lesions, neurologic involvement) were small, limiting power to detect small differences by accident type. Initial visual acuity, open- versus closed-globe injury classification, acute traumatic optic neuropathy, and details of acute surgical treatment and visual rehabilitation were not uniformly recorded and therefore could not be analyzed as determinants of the final visual outcome. Important behavioral/environmental variables (mechanism, impact direction, seatbelt/helmet use) were incompletely documented, limiting prevention-focused analyses. Finally, quality-of-life, psychosocial outcomes, and long-term rehabilitation were outside the study scope. Prospective, multicenter studies with standardized capture of protective-equipment use and timing of assessments are needed to refine risk estimates and guide prevention.

The modest sample size and the small numbers of patients within some visual-impairment categories precluded reliable multivariable modeling. Therefore, the reported associations are unadjusted and should not be interpreted as independent predictive effects. Because multiple exploratory comparisons were performed without a global multiplicity correction, an increased risk of type I error cannot be excluded. In particular, nominal associations with *p* values close to 0.05 should be regarded as hypothesis-generating and require confirmation in larger cohorts. Although the sample included all eligible cases during the study period and was sufficient for the descriptive objectives of the study, it limited the statistical power and precision of the subgroup comparisons. Therefore, associations based on small subgroups may change in magnitude or statistical significance in larger cohorts and should be interpreted as exploratory.

The cohort included only patients who had completed treatment and were referred or presented for forensic medical evaluation. This introduces selection and referral bias because patients who did not undergo medico-legal assessment and those still receiving active treatment were not represented; consequently, the findings are generalizable primarily to similar post-healing medico-legal populations rather than to the full spectrum of traffic-related ocular trauma.

The interval between the accident and the final forensic/ophthalmological assessment was not systematically recorded in the study dataset and therefore could not be summarized. Consequently, potential variability in the timing of post-treatment assessment could not be evaluated. In addition, the initial severity of ocular trauma and details of the treatments received were not uniformly available in the retrospective records. Consequently, the extent to which post-healing outcomes reflected injury severity rather than differences in management could not be determined. Prospective studies using standardized documentation of injury characteristics, treatment, and follow-up timing would allow these effects to be evaluated more reliably.

## 5. Conclusions

In this retrospective medico-legal cohort of traffic accident-related ocular injuries, severe/total visual impairment was associated with optic atrophy and intracranial bleeding, while the observed increase in disability percentages with impairment severity was consistent with the structure of the regulatory scoring system. Although the study did not detect a statistically significant association between accident type and visual-impairment severity, this finding should not be interpreted as evidence of no association; the comparison remains exploratory and hypothesis-generating because of the limited sample size and small subgroups. These findings may inform multidisciplinary post-healing evaluation using standardized visual metrics and targeted neurologic assessment; however, the study did not directly evaluate whether this approach improves the consistency or defensibility of disability determinations.

## Figures and Tables

**Table 1:** Demographic, accident-related, and medico-legal characteristics of the patients.

Variable	
Disability rate, mean ± SD	19.94 ± 20.63
Age (years), mean ± SD	32.65 ± 15.82
LogMAR, mean ± SD	0.72 ± 1.03
Gender	
- Female, *n* (%)	31 (41.3)
- Male, *n* (%)	44 (58.7)
Accident type	
- Within-vehicle, *n* (%)	34 (45.3)
- Outside-the-vehicle, *n* (%)	18 (24.0)
- Motorcycle, *n* (%)	23 (30.7)
Orbital fracture, *n* (%)	15 (20.0)

*n*: number; SD: standard deviation; LogMAR: logarithm of the minimum angle of resolution.

**Table 2:** Distribution of post-healing ocular and associated clinical findings.

Variable	
Neuro-ophthalmological involvements, *n* (%)	7 (9.3)
- Cranial nerve damage, *n* (%)	4 (5.3)
- RNFL thinning, *n* (%)	1 (1.3)
- Central vision loss, *n* (%)	2 (2.7)
- Hemianopsia, *n* (%)	1 (1.3)
- Optic atrophy, *n* (%)	20 (26.7)
Anterior-segment involvements, *n* (%)	15 (20.0)
- Corneal scar/graft/laceration, *n* (%)	8 (10.7)
- Aniridia, *n* (%)	2 (2.7)
- Aphakia, *n* (%)	2 (2.7)
- Iridodialysis, *n* (%)	2 (2.7)
- Scleral scar, *n* (%)	2 (2.7)
- Traumatic cataract, *n* (%)	1 (1.3)
- Occlusopupil, *n* (%)	1 (1.3)
- Pupil irregularity, *n* (%)	1 (1.3)
- Myotic fundus, *n* (%)	1 (1.3)
- Punctate epitheliopathy, *n* (%)	1 (1.3)
- Uveal prolapsus, *n* (%)	1 (1.3)
- Sclera perforation, *n* (%)	1 (1.3)
- Cornea perforation, *n* (%)	1 (1.3)
- Leukoma, *n* (%)	1 (1.3)
Posterior-segment involvements, *n* (%)	10 (13.3)
- Retinal/subretinal hemorrhage, *n* (%)	3 (4.0)
- Commotio retinae sequelae, *n* (%)	3 (4.0)
- Vitreus/retina detachment, *n* (%)	2 (2.7)
- Pigment epithelial degeneration, *n* (%)	2 (2.7)
- Siliconized retina, *n* (%)	1 (1.3)
- Fovea damage, *n* (%)	1 (1.3)
- Choroid rupture, *n* (%)	1 (1.3)
Eyelid and periorbital lesion, *n* (%)	31 (41.3)
- Eyelid scar, *n* (%)	15 (20.0)
- Ptosis, *n* (%)	7 (9.3)
- Lagophthalmia, *n* (%)	6 (8.0)
- Epiphora, *n* (%)	3 (4.0)
- Orbital compartment syndrome, *n* (%)	2 (2.7)
- Conjunctiva scar/laceration, *n* (%)	2 (2.7)
- Enophthalmia, *n* (%)	2 (2.7)
- Entropion, *n* (%)	2 (2.7)
- Eyelash loss, *n* (%)	1 (1.3)
- Canalicular laceration, *n* (%)	1 (1.3)
- Foreign body, *n* (%)	1 (1.3)
- Chalazion, *n* (%)	1 (1.3)
Eye movement problems, *n* (%)	24 (32.0)
- Paralytic strabismus, *n* (%)	1 (1.3)
- Restrictive strabismus, *n* (%)	5 (6.7)
- Diplopia, *n* (%)	21 (28.0)
- Compensatory head position, *n* (%)	4 (5.3)
- Exotropia, *n* (%)	3 (4.0)
- Hypertropia, *n* (%)	3 (4.0)
- Combined strabismus, *n* (%)	1 (1.3)
- Nystagmus, *n* (%)	1 (1.3)
- Intracranial bleeding, *n* (%)	12 (16.0)

More than one involvement was present in some cases. *n*: number; RNFL: retinal nerve fiber layer.

**Table 3:** Relationships of demographic and general clinical characteristics with accident type.

	Within-Vehicle	Outside-the- Vehicle	Motorcycle	
Disability rate (mean ± SD)	19.29 ± 20.14	22.88 ± 20.92	18.60 ± 21.79	0.554 *
Age (years) (mean ± SD)	35.29 ± 17.58	30.50 ± 16.55	30.43 ± 12.13	0.426 **
Visual acuity (LogMAR ± SD)	0.93 ± 1.18	0.61 ± 1.00	0.49 ± 0.77	0.573
Gender				0.001
- Female, *n* (%)	19 (55.9) ^a^	10 (55.6) ^a^	2 (8.7) ^b^	
- Male, *n* (%)	15 (44.1) ^a^	8 (44.4) ^a^	21 (91.3) ^b^	
Orbital fracture, *n* (%)	5 (14.7)	6 (33.3)	4 (17.4)	0.320
Neuro-ophthalmic involvements, *n* (%)	3 (8.8)	2 (11.1)	2 (8.7)	1.000
- Cranial nerve damage, *n* (%)	1 (2.9)	1 (5.6)	2 (8.7)	
- Central vision loss, *n* (%)	2 (5.9)	0 (0.0)	0 (0.0)	
- Optic atrophy, *n* (%)	6 (17.6)	7 (38.9)	7 (30.4)	0.228

*n*: number; SD: standard deviation; LogMAR: logarithm of the minimum angle of resolution. Different superscript letters indicate statistically significant pairwise differences. * Kruskal–Wallis test; ** one-way ANOVA test. Categorical variables were analyzed using Pearson’s chi-square test or the Fisher–Freeman–Halton exact test with Monte Carlo estimation, as appropriate. Reported *p* values are nominal unless a Bonferroni-adjusted post hoc comparison is specifically indicated. Percentages are column percentages within accident type.

**Table 4:** Relationships of detailed ocular examination findings with accident type.

	Within-Vehicle	Outside-the-Vehicle	Motorcycle	
Anterior-segment involvement, *n* (%)	9 (26.5)	2 (11.1)	4 (17.4)	0.398
- Corneal scar/graft/laceration, *n* (%)	6 (17.6)	0 (0.0)	2 (8.7)	0.161
- Aniridia, *n* (%)	1 (2.9)	1 (5.6)	0 (0.0)	
- Aphakia, *n* (%)	1 (2.9)	1 (5.6)	0 (0.0)	
- Iridoliasis, *n* (%)	2 (5.9)	0 (0.0)	0 (0.0)	
- Scleral scar, *n* (%)	0 (0.0)	0 (0.0)	2 (8.7)	
- Traumatic cataract, *n* (%)	1 (2.9)	0 (0.0)	0 (0.0)	
- Occlusopupil, *n* (%)	1 (2.9)	0 (0.0)	0 (0.0)	
- Pupil irregularity, *n* (%)	1 (2.9)	0 (0.0)	0 (0.0)	
- Myotic fundus, *n* (%)	1 (2.9)	0 (0.0)	0 (0.0)	
- Punctate epitheliopathy, *n* (%)	0 (0.0)	0 (0.0)	1 (4.3)	
- Uveal prolapsus, *n* (%)	1 (2.9)	0 (0.0)	0 (0.0)	
- Sclera perforation, *n* (%)	1 (2.9)	0 (0.0)	0 (0.0)	
- Cornea perforation, *n* (%)	1 (2.9)	0 (0.0)	0 (0.0)	
- Corneal leukoma, *n* (%)	0 (0.0)	0 (0.0)	1 (4.3)	
Posterior-segment involvement, *n* (%)	7 (20.6)	1 (5.6)	2 (8.7)	0.276
- Retinal/subretinal hemorrhage, *n* (%)	2 (5.9)	0 (0.0)	1 (4.3)	
- Commotio retinae sequelae, *n* (%)	2 (5.9)	0 (0.0)	1 (4.3)	
- Vitreus/retina detachment, *n* (%)	1 (2.9)	1 (5.6)	0 (0.0)	
- Retinal pigment epithelial degeneration, *n* (%)	2 (5.9)	0 (0.0)	0 (0.0)	
- Siliconized retina, *n* (%)	1 (2.9)	0 (0.0)	0 (0.0)	
- Fovea damage, *n* (%)	1 (2.9)	0 (0.0)	0 (0.0)	
- Choroid rupture, *n* (%)	0 (0.0)	0 (0.0)	1 (4.3)	
Eyelid and periorbital lesion, *n* (%)	13 (38.2)	5 (27.8)	13 (56.5)	0.158
- Eyelid scar, *n* (%)	9 (26.5)	2 (11.1)	4 (17.4)	0.396
- Ptosis, *n* (%)	2 (5.9)	2 (11.1)	3 (13.0)	0.597
- Lagophthalmia, *n* (%)	2 (5.9)	0 (0.0)	4 (17.4)	0.107
- Epiphora, *n* (%)	0 (0.0)	1 (5.6)	2 (8.7)	
- Orbital compartment syndrome, *n* (%)	0 (0.0)	1 (5.6)	1 (4.3)	
- Conjunctiva scar/laceration, *n* (%)	1 (2.9)	0 (0.0)	1 (4.3)	
- Enophthalmia, *n* (%)	2 (5.9)	0 (0.0)	0 (0.0)	
- Entropion, *n* (%)	2 (5.9)	0 (0.0)	0 (0.0)	
- Eyelash loss, *n* (%)	1 (2.9)	0 (0.0)	0 (0.0)	
- Canalicula laceration, *n* (%)	0 (0.0)	0 (0.0)	1 (4.3)	
- Intraocular foreign body, *n* (%)	1 (2.9)	0 (0.0)	0 (0.0)	
Eye movement problems, *n* (%)	10 (29.4)	9 (50.0)	5 (21.7)	0.142
- Paralytic strabismus	1 (2.9)	0 (0.0)	0 (0.0)	
- Restrictive strabismus	2 (5.9)	1 (5.6)	2 (8.7)	1.000
- Diplopia	9 (26.5)	8 (44.4)	4 (17.4)	0.154
- Compensatory head position	2 (5.9)	2 (11.1)	0 (0.0)	
- Nystagmus	0 (0.0)	1 (5.6)	0 (0.0)	
- Hemianopsia	0 (0.0)	1 (5.6)	0 (0.0)	
- Intracranial bleeding	5 (14.7)	3 (16.7)	4 (17.4)	1.000
- None	29 (85.3)	15 (83.3)	19 (82.6)	

More than one involvement was present in some cases. *n*: number; findings observed in fewer than five patients are presented descriptively and were not subjected to inferential comparison. Categorical variables were analyzed using Pearson’s chi-square test or the Fisher–Freeman–Halton exact test with Monte Carlo estimation, as appropriate. Percentages are column percentages within accident type.

**Table 5:** Relationships of demographic and clinical findings with visual-impairment severity.

	None	Mild	Moderate	Severe/Total	
	Mean ± SD	Mean ± SD	Mean ± SD	Mean ± SD	*p*
Disability rate	10.00 ± 10.04 ^a^	12.90 ± 12.04 ^a^	19.57 ± 16.65 ^a.b^	42.50 ± 23.35 ^b^	<0.001
Age (years)	28.97 ± 14.74	39.70 ± 17.83	34.85 ± 15.69	35.35 ± 16.16	0.191
	*n* (%)	*n* (%)	*n* (%)	*n* (%)	*p*
Gender					0.402
Female	13 (41.9)	6 (19.4)	4 (12.9)	8 (25.8)	
Male	25 (56.8)	4 (9.1)	3 (6.8)	12 (27.3)	
Accident type					0.682
Within-vehicle	17 (50.0)	2 (5.9)	4 (11.8)	11 (32.4)	
Outside-the-vehicle	9 (50.0)	4 (22.2)	1 (5.6)	4 (22.2)	
Motorcycle	12 (52.2)	4 (17.4)	2 (8.7)	5 (21.7)	
Orbital fracture					0.072
Present	12 (80.0)	1 (6.7)	1 (6.7)	1 (6.7)	
Absent	26 (44.1)	9 (15.3)	5 (8.5)	19 (32.2)	
Neuro-ophthalmological involvement					0.505
Present	3 (42.9)	0 (0.0)	1 (14.3)	3 (42.9)	
Absent	35 (51.5)	10 (14.7)	6 (8.8)	17 (25.0)	
Optic atrophy					<0.001
Present	1 (5.0)	5 (25.0)	3 (15.0)	11 (55.0)	
Absent	37 (67.3)	5 (9.1)	4 (7.3)	9 (16.4)	
Anterior segment lesion					0.365
Present	5 (33.3)	2 (13.3)	2 (13.3)	6 (40.0)	
Absent	33 (55.0)	8 (13.3)	5 (8.3)	14 (23.3)	
Posterior segment lesion					0.105
Present	2 (20.0)	2 (20.0)	2 (20.0)	4 (40.0)	
Absent	36 (55.4)	8 (12.3)	5 (7.7)	16 (24.6)	
Eyelid and periorbital lesion					0.024
Present	21 (67.7)	5 (16.1)	1 (3.2)	4 (12.9)	
Absent	17 (38.6)	5 (11.4)	6 (13.6)	16 (36.4)	
Eye movement examination findings					0.045
Present	17 (70.8)	3 (12.5)	2 (8.3)	2 (8.3)	
Absent	21 (41.2)	7 (13.7)	5 (9.8)	18 (35.3)	
Compensatory head position					
Present	2 (50.0)	1 (25.0)	0 (0.0)	1 (25.0)	
Absent	36 (50.7)	9 (12.7)	7 (9.9)	19 (26.8)	
Intracranial bleeding					0.007
Present	2 (16.7)	1 (8.3)	1 (8.3)	8 (66.7)	
Absent	36 (57.1)	9 (14.3)	6 (9.5)	12 (19.0)	

More than one involvement was present in some cases. *n*: number; SD: standard deviation. Different superscript letters indicate statistically significant pairwise differences. Categorical variables were analyzed using Pearson’s chi-square test or the Fisher–Freeman–Halton exact test with Monte Carlo estimation, as appropriate. Percentages are row percentages across visual-impairment categories. Different superscript letters indicate statistically significant Bonferroni-adjusted post hoc differences. *p* values reported for categorical variables are omnibus tests across the four visual-impairment categories; category-specific post hoc comparisons were not performed.

## Data Availability

Data underlying this article will be shared on reasonable request to the corresponding author. Individual-level data are anonymized and subject to institutional restrictions.

## References

[1] Li C., Fu Y., Liu S., Yu H., Yang X., Zhang M., Liu L. (2023). The global incidence and disability of eye injury: An analysis from the Global Burden of Disease Study 2019. EClinicalMedicine.

[2] Bhatnagar N.V., Uppuluri A., Bhagat N., Langer P.D. (2024). Epidemiology of motor vehicle accident-associated ocular trauma. Int. Ophthalmol..

[3] Kuhn F., Morris R., Witherspoon C.D., Mann L. (2006). Epidemiology of blinding trauma in the United States Eye Injury Registry. Ophthalmic Epidemiol..

[4] McGwin G., Owsley C. (2005). Risk factors for motor vehicle collision-related eye injuries. Arch. Ophthalmol..

[5] Aires C.C.G., Araújo H.T., Souza R.R.L., Santos A.J.F.D., Vasconcellos R.J.H., Vasconcelos B.C.D.E. (2023). Relationship between the use and types of helmets with facial injuries: A prospective study. Rev. Col. Bras. Cir..

[6] May D.R., Kuhn F.P., Morris R.E., Witherspoon C.D., Danis R.P., Matthews G.P., Mann L. (2000). The epidemiology of serious eye injuries from the United States Eye Injury Registry. Graefes Arch. Clin. Exp. Ophthalmol..

[7] Rao S.K., Greenberg P.B., Filippopoulos T., Scott I.U., Katsoulakis N.P., Enzer Y.R. (2008). Potential impact of seatbelt use on the spectrum of ocular injuries and visual acuity outcomes after motor vehicle accidents with airbag deployment. Ophthalmology.

[8] Sahu S.K., Radhakrishnan R.V., Mohanty C.R., Parija S., Palanisamy S., Mishra P., Sadangi D. (2024). Pattern and clinical profile of patients with ocular trauma presenting to the emergency department of a teaching hospital in India: A prospective observational study. Turk. J. Emerg. Med..

[9] MacEwen C.J. (1989). Eye injuries: A prospective survey of 5671 cases. Br. J. Ophthalmol..

[10] Oner A., Kekec Z., Karakucuk S., Ikizceli I., Sozuer E.M. (2006). Ocular trauma in Turkey: A 2-year prospective study. Adv. Ther..

[11] Kyriakaki E.D., Symvoulakis E.K., Chlouverakis G., Detorakis E.T. (2021). Causes, occupational risk and socio-economic determinants of eye injuries: A literature review. Med. Pharm. Rep..

[12] Nalukenge C., Sebabi F.O., Okello B., Ntende J., Nakiyingi L., Nakanjako D., Nakubulwa F., Kalinaki A., Mulinde B., A Musika A. (2023). Factors associated with ocular injuries among adult road traffic accident patients presenting at Mulago National Referral Hospital, Uganda. Afr. Health Sci..

[13] Araújo T.A.C., Melo C.R.O., Mendes L.P., Higino T.M.M., Rocha C.S.D., Sabino L.P.F., Santos M.B. (2021). Analysis of ocular emergencies in a reference eye center in Brazil. Arq. Bras. Oftalmol..

[14] Al-Qurainy I.A., Stassen L.F., Dutton G.N., Stassen L., Moos K., Ei-Attar A. (1991). The characteristics of midfacial fractures and the association with ocular injury: A prospective study. Br. J. Oral. Maxillofac. Surg..

[15] Moussa G., Bassilious K., Mathews N. (2021). A novel Excel sheet conversion tool from Snellen fraction to LogMAR including ‘counting fingers’, ‘hand movement’, ‘light perception’ and ‘no light perception’ and focused review of literature of low visual acuity reference values. Acta Ophthalmol..

[16] T.C. Sağlık Bakanlığı (2019). Erişkinler İçin Engellilik Değerlendirmesi Hakkında Yönetmelik. Resmî Gazete.

[17] Savaş Ç., Aras N., Yeşilbalkan T., Can İ.Ö. (2025). Post-traumatic visual sequelae from a forensic medicine perspective: A retrospective analysis of 10 years of data. Ulus Travma Acil Cerrahi Derg..

[18] Clevenger L.M., Cao J.L., Steinkerchner M.S., Nowacki A.S., Yuan A. (2024). Demographics, presenting features, and outcomes of adult patients with ocular trauma. J. Ophthalmol..

[19] Yadav N.K., Sami I., Priya P., Arya A.K., Abbasi S., Srivastava S., Chhabra S. (2026). Global patterns and predictors of oculofacial trauma: A systematic review and meta-analysis of the last two decades. IP Int. J. Ocul. Oncol. Oculoplasty..

[20] Saha I., Varshney A. (2026). Epidemiology of posterior segment trauma following closed-globe injury in Eastern Uttar Pradesh. Indian J. Public Health Res. Dev..

[21] Hashim Z.A., Mohammed S.K., Abdulla M.Y., Fawzi H.A. (2024). Presentations and incidence of ocular injuries caused by motorcycle accidents in Iraq. F1000Research.

[22] Lev Ari O., Shaked G., Michael T., Givon A., Bodas M., Israel Trauma Group (2022). Ocular injuries associated with two-wheeled electric transportation devices and motorcycle accidents. Sci. Rep..

[23] Gagrai J., Prasad R., Bhagat V., Kumari S., Mishra N. (2025). A study on the pattern of ocular injuries and their visual outcomes following road traffic accidents. Cureus.

[24] Ramani S., Thomas A., Kulkarni A.S. (2024). Ocular injuries in two-wheeler-associated road traffic accidents. Ophthalmol. J..

[25] Zhong E., Chou T.Y., Chaleff A.J., Scofield-Kaplan S.M., Perzia B.M., Naqvi J., Hou W. (2022). Orbital fractures and risk factors for ocular injury. Clin. Ophthalmol..

[26] Abhirrami M., Parthibavijayan R., Baratan S. (2025). Through the eyes of trauma—Understanding ocular injuries and orbital fractures in road traffic accidents. TNOA J. Ophthalmic Sci. Res..

[27] Turadagi K., Bellary R., Channashetti G. (2025). Ophthalmic implications following orbital trauma. Int. J. Res. Med. Sci..

[28] Frederick M., Kumaresan V., Anuradha P. (2023). A clinical study on types of ocular injuries following road traffic accidents in a tertiary health care hospital in Chennai, Tamil Nadu. Int. J. Adv. Med..

[29] Al Amry M., AlHijji L., Elkhamary S.M., Mousa A., AlGaeed A., AlGhadeer H. (2023). Optic nerve avulsion: Pattern and etiologies at a tertiary eye care center in Saudi Arabia: An 8-year retrospective study. Clin. Ophthalmol..

[30] Sujithra H., Shah K., Greeshma C. (2023). Clinical profile and visual outcome in patients with traumatic optic neuropathy. Indian J. Ophthalmol..

[31] Gurung J., Karn M., Gurung P.K., Sapkota S. (2024). Neuro-ophthalmic manifestations in traumatic brain injury: A hospital-based study from Nepal. Ann. Med. Surg..

[32] Hârtie V.L., Anton N., Boișteanu O., Comanescu M.P., Pătrășcanu E., Bologa E., Șulea D., Danielescu C., Turliuc M.D. (2025). Traumatic brain injury and its ophthalmologic implications. Rom. J. Ophthalmol..

[33] Almutairi N.M. (2025). Visual dysfunctions in mild traumatic brain injury: A focus on accommodative system impairments. Life.

[34] Kim E.J., Ganga A., Kang C., Elnemer W., Lee J.Y., Ronquillo Y.C., Hoopes P.C., Moshirfar M. (2022). Motorcycle-associated ocular injuries: A narrative review. Clin. Ophthalmol..

[35] Sharma B., Agcon A.M.B., Agriantonis G., Kiernan S.R., Bhatia N.D., Twelker K., Shafaee Z., Whittington J. (2024). Patterns of brain injury and clinical outcomes related to trauma from collisions involving motor vehicles. J. Clin. Med..

[36] Nutbeam T., Weekes L., Heidari S., Fenwick R., Bouamra O., Smith J., Stassen W. (2022). Sex-disaggregated analysis of the injury patterns, outcome data and trapped status of major trauma patients injured in motor vehicle collisions. BMJ Open.

[37] Finger R.P., Fenwick E., Marella M., Dirani M., Holz F.G., Chiang P.P.C., Lamoureux E.L. (2011). The impact of vision impairment on vision-specific quality of life in Germany. Investig. Ophthalmol. Vis. Sci..

